# The Inhibitory Effects of Low-Dose Ionizing Radiation in IgE-Mediated Allergic Responses

**DOI:** 10.1371/journal.pone.0136394

**Published:** 2015-08-28

**Authors:** Hae Mi Joo, Su Jin Kang, Seon Young Nam, Kwang Hee Yang, Cha Soon Kim, In Kyung Lee, Ji Young Kim

**Affiliations:** Low-dose Radiation Research Team, Radiation Health Institute, Korea Hydro & Nuclear Power Co., Ltd., Seoul, Korea; Cincinnati Children's Hospital Medical Center, University of Cincinnati College of Medicine, UNITED STATES

## Abstract

Ionizing radiation has different biological effects according to dose and dose rate. In particular, the biological effect of low-dose radiation is unclear. Low-dose whole-body gamma irradiation activates immune responses in several ways. However, the effects and mechanism of low-dose radiation on allergic responses remain poorly understood. Previously, we reported that low-dose ionizing radiation inhibits mediator release in IgE-mediated RBL-2H3 mast cell activation. In this study, to have any physiological relevance, we investigated whether low-dose radiation inhibits allergic responses in activated human mast cells (HMC-1(5C6) and LAD2 cells), mouse models of passive cutaneous anaphylaxis and the late-phase cutaneous response. High-dose radiation induced cell death, but low-dose ionizing radiation of <0.5 Gy did not induce mast cell death. Low-dose ionizing radiation that did not induce cell death significantly suppressed mediator release from human mast cells (HMC-1(5C6) and LAD2 cells) that were activated by antigen-antibody reaction. To determine the inhibitory mechanism of mediator released by low-dose ionizing radiation, we examined the phosphorylation of intracellular signaling molecules such as Lyn, Syk, phospholipase Cγ, and protein kinase C, as well as the intracellular free Ca^2+^ concentration ([Ca^2+^]_i_). The phosphorylation of signaling molecules and [Ca^2+^]_i_ following stimulation of FcεRI receptors was inhibited by low dose ionizing radiation. In agreement with its *in vitro* effect, ionizing radiation also significantly inhibited inflammatory cells infiltration, cytokine mRNA expression (TNF-α, IL-4, IL-13), and symptoms of passive cutaneous anaphylaxis reaction and the late-phase cutaneous response in anti-dinitrophenyl IgE-sensitized mice. These results indicate that ionizing radiation inhibits both mast cell-mediated immediate- and delayed-type allergic reactions *in vivo* and *in vitro*.

## Introduction

Since radiation was discovered, it has been used in a wide variety of areas including medical diagnosis, therapy, and industry. However, the effects of radiation on humans are dualistic in nature and depend on the cell type, radiation dose, and post-irradiation time point, and can be a health hazard if used improperly [1, 2]. For example, although the immune-suppressing effect of high-dose radiation has been clearly demonstrated [3, 4], low-dose irradiation activates the immune system in several ways, leading in some cases to faster wound healing and increased resistance to toxins, infections, and tumor cell injection [1]. For the first four decades of the last century, low-dose irradiation was the recommended treatment for many human diseases [5, 6, 7].

Mast cells are key effector cells in IgE-mediated immune responses [8, 9] and elicit immediate-type hypersensitivity reactions via a degranulation response to antigen cross-linking of FcεRI followed by release of pro-inflammatory mediators such as histamine, leukotrienes, prostaglandins, and chemokines [9]. These mediators recruit T lymphocytes and granulocytic inflammatory cells, increases vascular permeability, and lead to smooth muscle contraction [10, 11].

Mast cell degranulation induced by FcεRI receptors involves multiple tyrosine phosphorylation events including activation of the Src family kinase Lyn, which in turn phosphorylates and activates Syk kinase [12]. Subsequent activation of PI3K and phospholipase Cγ (PLCγ) induces a Ca^2+^ efflux from the endoplasmic reticulum. Increased cytosolic [Ca^2+^] promotes exocytosis and mast cell degranulation [13].

Previous studies have reported that mice passively sensitized with anti-dinitrophenol (DNP) IgE antibody exhibit an IgE-mediated cutaneous reaction with an immediate-phase response at 1 h and a late-phase response at 24 h after a challenge with dinitrofluorobenzene (DNFB) [14–17]. In many patients with allergies, intradermal challenge with a specific antigen or anti-IgE induces an immediate wheal and flare reaction that is followed 4–8 h later by a period of persistent swelling and leukocyte infiltration termed the late-phase cutaneous reaction [18–19].

We recently reported that low-dose radiation inhibits release of mediators such as histamine, β-hexosaminidase, and cytokines from RBL-2H3 mast cells with IgE-mediated signaling suppression due to reduction of FcεRI receptor expression [20], an observation that formed the basis of the hypothesis for the work presented in this study. We hypothesized that low-dose radiation directly affects mast cells and influences the allergic response via antigen-antibody reaction. Therefore, to investigate any physiological relevance between anti-allergic effects and low-dose ionizing radiation, this study was conducted in human mast cells and a mouse model of typical cutaneous or late-phase allergic dermatitis.

## Materials and Methods

### Cell culture

The HMC-1(5C6) and LAD2 cells were established at 1996 by Dr. BM Henz [21] and at 2003 by Dr. AS Kirshenbaum [22] separately, since than used widely as human mast cell line.

The HMC-1(5C6) cell line was kindly provided by Dr. T Zuberbier (Universitatsmedizin, Berlin). HMC-1(5C6) cells were grown in Iscove’s Modified Dulbecco’s Medium (GIBCO, Grand Island, NY) containing 10% FBS (GIBCO), 100 U/ml penicillin, and 100 μg/ml streptomycin (GIBCO) [21].

The human mast cell line LAD-2 was kindly provided by Dr. AS Kirshenbaum (NIAID, Bethesda, MD). LAD2 cells were cultured in StemPro-34 serum-free medium (GIBCO) supplemented with 2 mM l-glutamine (GIBCO) and 100 ng/ml recombinant human SCF (R&D Systems, Inc., Minneapolis, MN) [22].

### Irradiation of cells

Cells and mice were irradiated with total doses ranging between 0.01 and 1 Gy using a ^137^Cs γ-irradiator (IBL 437C, CIS Bio International, Bangnols sur Ceze, France) with a dose rate of 0.8 Gy/min.

### Cell survival measurements

Cell viability was measured using MTT (Sigma, St. Louis, MO) 72 h after irradiation. Yellow MTT is reduced to purple formazan in the mitochondria of living cells. The absorbance of this colored solution was measured at 540 nm with a spectrophotometer (Lab System, Helsinki, Finland) [23]. For long-term cell survival determination, serially diluted irradiated cells were seeded in 35-mm dishes containing methylcellulose complete medium (R&D Systems, Inc.). After incubation for 14 days, colonies were stained with nitro blue tetrazolium (Sigma). Colonies containing more than 50 cells were counted. Survival fractions were calculated by dividing the mean colony count for each radiation dose by the mean colony count of the non-irradiated control group. Survival curves were plotted as the log of the survival fraction of cells versus the radiation dose.

### Assays for histamine and β-hexosaminidase secretion

HMC-1(5C6) and LAD2 cells were separately sensitized with 0.5 μg/ml myeloma IgE (Calbiochem, La Jolla, CA) and 0.1 μg/ml biotinylated IgE (Antibodyshop, Gentofte, Denmark). Cells were washed with modified Tyrode’s buffer, irradiated with a total dose ranging from 0.01 to 1 Gy, and stimulated with 1 μg/ml anti-IgE (Dako, Glostrup, Denmark) and 0.01 μg/ml streptavidin (Sigma). After 1 h, reactions were terminated by placing the plates in an ice bath. Histamine and *β*-hexosaminidase were measured as previously described [20].

### Cytokine measurements

IgE-sensitized HMC-1(5C6) and LAD2 cells were irradiated and stimulated with antigen for 5 h. The concentration of tumor necrosis factor alpha (TNF-α) in the cell culture supernatant was measured using ELISA kit (R&D Systems, Inc.).

### Measurement of intracellular Ca^2+^ levels

Cytosolic calcium levels were measured using the calcium-reactive fluorescence probe Fluo-3/AM (Molecular Probes, Eugene, OR). Briefly, HMC-1(5C6) and LAD2 cells were sensitized with 0.5 μg/ml myeloma IgE (Calbiochem) and 0.1 μg/ml biotinylated IgE (Antibodyshop). Cells were irradiated and incubated with 5 μM Fluo-3/AM in modified Tyrode’s buffer (without 1 mM CaCl_2_) for 30 min at 37°C. After washing, cells were stimulated with 1 μg/ml anti-IgE (Dako) and 0.01 μg/ml streptavidin (Sigma). Fluo-3/AM fluorescence intensities were monitored at 15-sec intervals for 15 min using a microplate fluorometer (Berthold Technologies, Bad Wildbad, Germany) with excitation and emission wavelengths of 485 and 535 nm, respectively. Intracellular calcium levels were measured as previously described [20].

### Immunoblotting

Human mast cells were harvested and lysed in a lysis buffer containing 20 mM Tris (pH 7.6), 150 mM NaCl, 1% Triton X-100, protease inhibitors cocktail, and phosphatase inhibitor cocktail (Thermo Scientific, Waltham, MA). Protein quantification was determined with the Bradford assay (Bio-Rad). Cell lysates of equal protein concentrations were prepared in SDS sample buffer (Invitrogen, Carlsbad, CA), separated with SDS-PAGE (10% or 12% acrylamide), and transferred to nitrocellulose membranes (Amersham Biosciences, Uppsala, Sweden). Membranes were blocked with 5% skim milk (GIBCO) in PBS containing 0.1% Tween 20 and incubated with the indicated primary antibodies for 3 h at room temperature or overnight at 4°C. After washing with PBS containing 0.1% Tween 20, membranes were incubated with secondary antibodies for 1 h at room temperature. Protein bands were visualized using ECL solution (Amersham Biosciences).

### Flow cytometry

HMC-1(5C6) and LAD2 cells were sensitized with 0.5 μg/ml myeloma IgE (Calbiochem) and 0.1 μg/ml biotinylated IgE (Antibodyshop), washed with PBS, and irradiated with a total dose ranging from 0.01 to 0.5 Gy. After a 30-min incubation, cells were treated with FITC-conjugated rat anti-IgE (BD Pharmingen, San Diego, CA) and analyzed with flow cytometry (Beckman Coulter, Inc., Krefeld, Germany).

### IgE-mediated passive cutaneous reaction

Six-week-old female C57BL/6 mice were obtained from the Shizuoka Laboratory Animal Cooperation (Hamamatsu, Japan) and maintained in pathogen-free conditions. All procedures were approved by the Institutional Animal Care and Use Committee at the Radiation Health Institute of Korea Hydro and Nuclear Power Company, and mice were treated in accordance with governmental guidelines and the guidelines of the Radiation Health Institute for the care of animals. The condition of the mice was checked every day. All treatment was performed under Zoletil 50 (25mg/kg) anesthesia. All mice were euthanized using an overdose of Zoletil 50 (50mg/kg) prior to collection and fixation of tissues.

Each mouse was primed to express an IgE-mediated passive cutaneous anaphylaxis reaction in the left ear. A control reaction was established in the right ear. Mice were lightly anesthetized with 25 mg/kg zoletil 50 (Virbac Laboratories) and sensitized with intradermal injection of 20 ng/10 μl anti-DNP IgE mAb (Sigma) into the left ear, whereas the right ear was given a saline injection as a control. The next day, mice were irradiated and given an intraveneous (i.v.) injection of 100 μg DNP-HSA (human serum albumin; Sigma) in 200 μl of 1% Evans blue dye (Sigma) to permit visualization of sites of increased vascular permeability. Thirty minutes later, an 8-mm ear punch was collected in 300 μl formamide and incubated at 80°C for 2 h in a water bath to extract the Evan’s blue dye. The absorbance was determined at 620 nm [24].

### IgE-mediated late-phase cutaneous reaction

The mice were given an i.v. injection of 2 μg anti-DNP IgE (Sigma). The next day, mice were irradiated with 0.01–0.5 Gy, and a skin reaction was elicited with the application of 20 μl of 0.3% DNFB solution in acetone/olive oil (4:1) to both sides of the left hind paw and left ear, and 20 μl of acetone/olive oil to the right hind paw and right ear as a control. The thickness of ear and hind paw was measured using a digital caliper (Mitutoyo Corporation, Japan). The weight of the ear punch (8 mm) and hind paw were also measured [14–17].

### Reverse Transcription Polymerase Chain Reaction (RT-PCR)

Total RNA was extracted from the mouse ear tissue using Trizol reagent (Ambion, Carlsbad, CA). A total of 10μg RNA was subjected to cDNA using the Iscript Advanced cDNA Synthesis Kit (BIO-RAD, Hercules, CA) according to the manufacturer’s instruction. The cDNAs were used for quantitative RT-PCR. The RT-PCR reaction mix was as follows: 10 μl cDNA (40 ng/μl), 1 μl forward primer, 1 μl reverse primer, 12 μl GoTaq qPCR master mix (Promega, WI, USA). PCR reactions were performed for 35 to 40 cycles in a 7500 Real-Time PCR System (Applied Biosystems, CA, USA). Each cycle consisted of the following steps: denaturation at 95°C for 20 sec, annealing at 60°C for 20 sec, and extension at 72°C for 45 sec. PCR products were analyzed using 1.2% or 2% agarose gels containing ethidium bromide staining. The band of PCR products was quantified by densitometry. Data were means of three independent experiments.

The primer sequences used for amplications were as follows: GAPDH (NM_001289726.1, 132bp) forward 5-AACTTTGGCATTGTGGAAGG-3, reverse 5-GGATGCAGGGATGATGTTCT-3; TNF-α (NM_001278601.1, 259bp) forward 5-CCACATCTCCCTCCAGAAAA-3, reverse 5-AGGGTCTGGGCCATAGAACT-3; IL-4 (NM_021283.2, 312bp) forward 5-TCAACCCCCAGCTAGTTGTC-3, reverse 5-ATCGAAAAGCCCGAAAGAGT-3; IL-13 (NM_008355.3, 54bp) forward 5-ATGCCCAACAAAGCAGAGAC-3, reverse 5-TGAGAGAACCAGGGAGCTGT-3. Primers were designed with Primer 3 software (http://primer3.ut.ee).

### Histology

Ears were removed and immediately immersed in 4% paraformaldehyde. The specimens were trimmed and fixed in this solution. Specimens were dehydrated in a graded ethanol series and embedded in paraffin. Then 4μm sections were cut and stained with hematoxylin and eosin, and toluidine for histological examination.

### Immunohistochemistry

To visualize infiltrating CD4 and CD8 cells, ear tissue specimens were blocked with 3% methanolic hydrogen peroxide (H_2_O_2_), treated with pepsin (Invitrogen, Carlsbad, CA) at 37°C for 10 min for antigen unmasking and blocked with 3% BSA. Specimen were subsequently incubated with primary antibody, Rat antibodies against mouse CD4 (BD Biosciences) and CD8 (BD Biosciences), visualized using an immunoperoxidase technique (BD Biosciences) and counterstained with hematoxylin.

FcεRI expression in ear tissue was incubated with rat antibody against mouse FcεRI labeled with FITC (BD Biosciences) and monitored by fluorescence microscopy (Carl Zeiss Meditec, Dublin, CA).

### Statistical analysis

Experimental data are presented as the mean ± the standard error of the mean (SEM). The analysis of the significance of differences between controls and experimental groups was performed using SAS software (version 8.1; SAS Institute Inc., Cary, NC) and *t*-tests. *p*-values less than 0.05 were considered significant.

## Results

### Effect of ionizing radiation on cell viability in human mast cells

**Fig 1:**
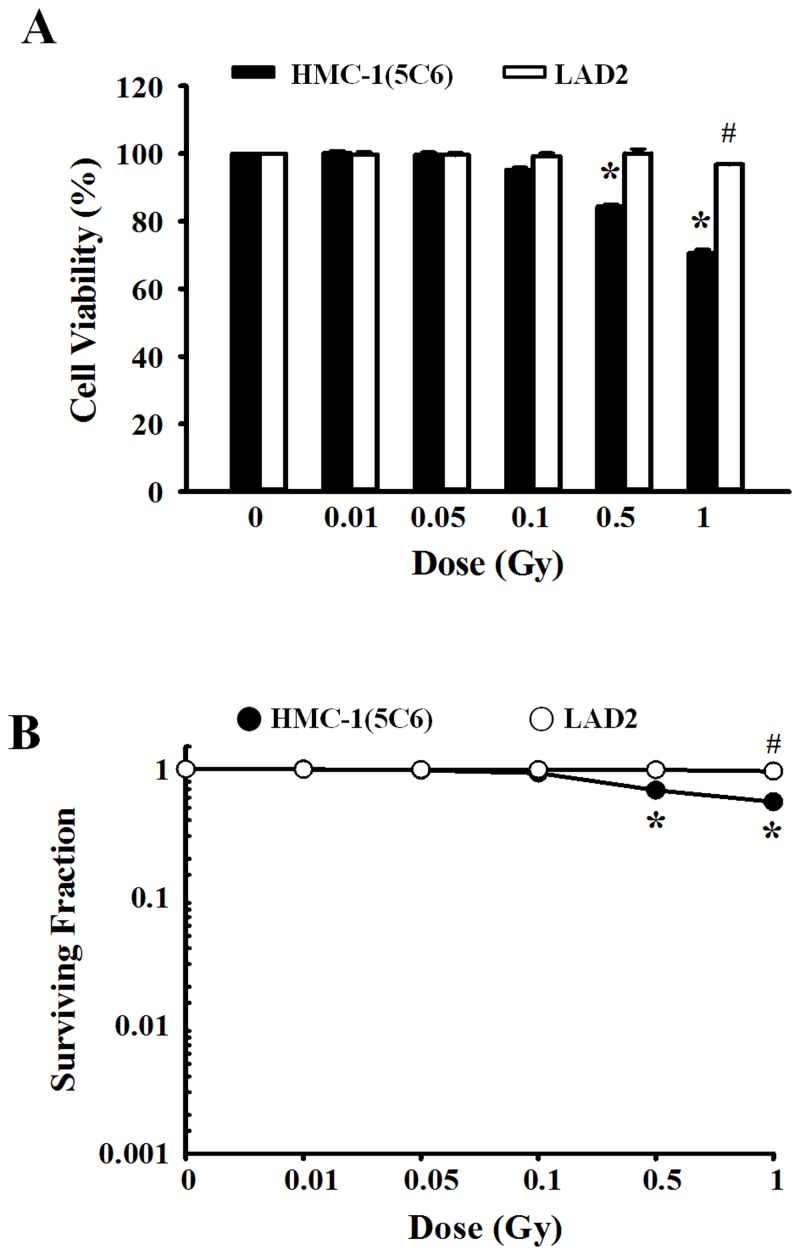
The effects of ionizing radiation on cell viability in human mast cells. HMC-1(5C6) and LAD2 cells were exposed to various doses (0–1 Gy) of ionizing radiation and incubated for 72 h (A) or 14 days (B). Cell survival was evaluated using MTT assays (A) and colony formation assays (B). Data represent the mean ± SEM from five independent experiments performed in duplicate. * p < 0.05, # p < 0.05 versus the corresponding control group of HMC-1(5C6) and LAD2 cells, respectively.

To examine the effect of ionizing radiation on cell viability, human mast cells (HMC-1(5C6) and LAD2 cells) were irradiated with 0 to 1 Gy and evaluated with the MTT assay (Fig 1A) to assess short-term viability and the colony formation assay to assess long-term viability (Fig 1B). Low-dose (<0.5 Gy) radiation did not show any effects on human mast cells, but high-dose radiation >0.5 Gy significantly reduced the viability of HMC-1(5C6) cells. For LAD2 cells, high-dose radiation (>1 Gy) induced cell death (Fig 1). LAD2 cells exhibited minimal radiation-induced cytotoxicity compared with HMC-1(5C6) cells.

### Effect of ionizing radiation on mediator release and cytokine production in human mast cells

**Fig 2:**
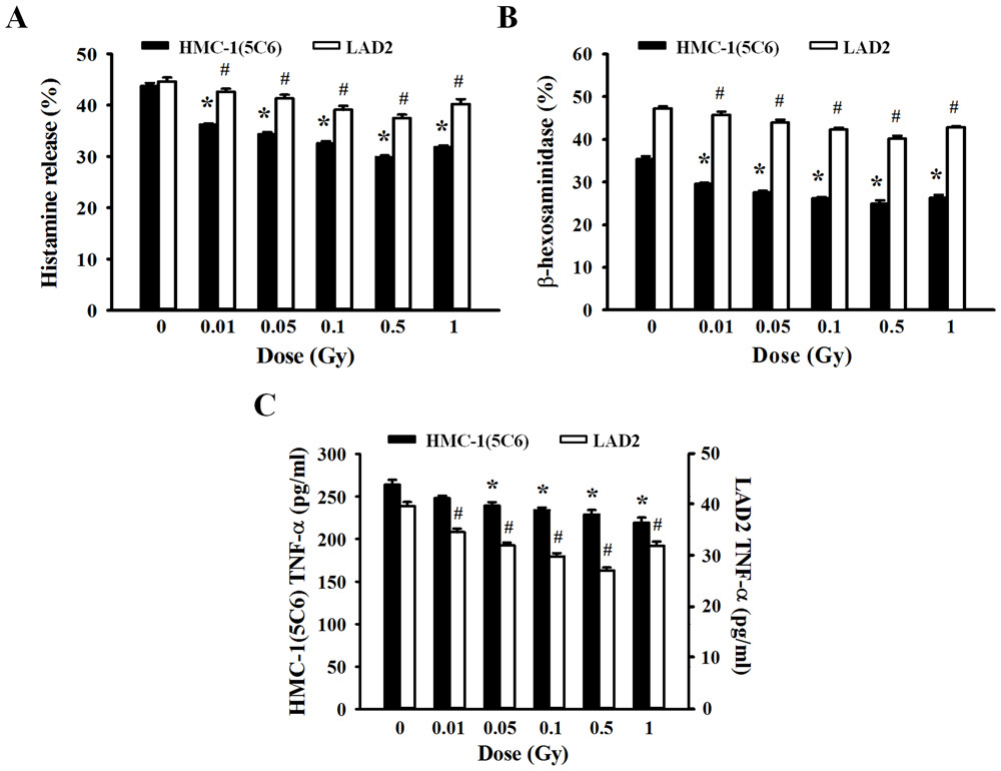
Inhibitory effects of radiation on mediator release and cytokine production by human mast cells. HMC-1(5C6) and LAD2 cells were incubated for 30 min in medium containing human IgE. Cells were exposed to ionizing radiation (0–1 Gy) before the addition of anti-IgE. The histamine concentration (A), β-hexosaminidase concentration (B), and cytokine (TNF-α) production (C) were determined in supernatants. Data represent the mean ± SEM from five independent experiments performed in duplicate. * p < 0.05, # p < 0.05 versus the corresponding control group of HMC-1(5C6) and LAD2 cells, respectively.

Mediator release by mast cells was used as a marker to evaluate mast cell activation. To examine whether ionizing radiation plays a role in IgE-dependent mast cell activation or regulates mediator release and inflammatory cytokine production, human mast cells (HMC-1(5C6) and LAD2 cells) were irradiated with different dose of ionizing radiation before a 30-min stimulation with antigen in anti-IgE sensitized cells and histamine, β-hexosaminidase, and TNF-α levels were determined. As shown in Fig 2, although antigen-activated human mast cells showed significantly elevated release of mediators, irradiated human mast cells showed a decrease in the levels of mediators released (histamine and β-hexosaminidase). In addition to mediator release by degranulation, IgE-mediated stimulation of mast cells induces production of inflammatory cytokines. To determine the effect of ionizing radiation on inflammatory cytokine production following activation of HMC-1(5C6) or LAD2 cells, we measured the TNF-α production in culture supernatants. Production of the inflammatory cytokine TNF-α was also inhibited by ionizing radiation in a dose-dependent manner (Fig 2C). These results demonstrate that ionizing radiation regulates mediator release and cytokine production in human mast cells.

IgE-mediated degranulation and cytokine production were suppressed by ionizing radiation in a dose-dependent manner but recovered somewhat with the 1-Gy dose. This phenomenon may be related to cell toxicity because high doses of ionizing radiation induced mast cell breakdown and the release of mediators. Therefore, we will not further examine the effects of irradiation exposure >1 Gy because both HMC-1(5C6) and LAD2 human mast cells showed cellular toxicity (Fig 1) and mast cell degranulation (Fig 2) following 1 Gy irradiation.

### Inhibitory effect of ionizing radiation on the FcεRI receptor-mediated signaling pathway

**Fig 3:**
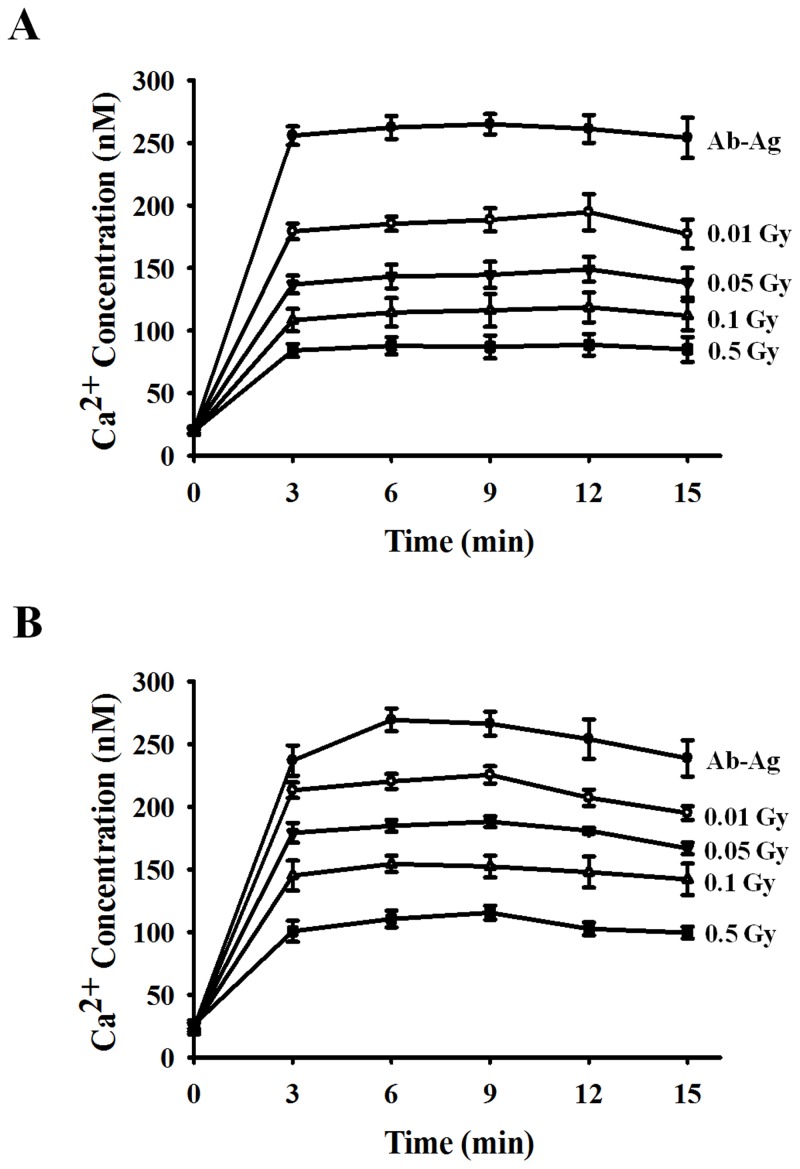
Effects of ionizing radiation on intracellular Ca^2+^ mobilization in activated human mast cells. HMC-1(5C6) (A) and LAD2 cells (B) were incubated for 30 min in medium containing human IgE, exposed to 0–0.5 Gy ionizing radiation, and incubated with 5 μM Fluo-3/AM fluorescence dye for 30 min. Fluo-3/AM-loaded cells were stimulated with anti-IgE and monitored at 15-sec intervals for 15 min. Data represent the mean ± SEM from five independent experiments performed in duplicate.

An aggregation of FcεRI on the surface of mast cells initiates a complex cascade of signaling events, with elevation of the intracellular calcium concentration ([Ca^2+^]_i_) as one of the critical common events. Therefore, we measured [Ca^2+^]_i_ using a Fluo-3/AM dye in human mast cells that were activated by the antigen-antibody reaction. As shown in Fig 3, as expected, an immediate and sustained [Ca^2+^]_i_ increase was observed in mast cells following antigen challenge. However, a sustained suppression of [Ca^2+^]_i_ was observed in both HMC-1 and LAD2 cells treated with ionizing radiation. In summary, intracellular calcium influx in human mast cells that were exposed to ionizing radiation in the range of 0.01 to 0.5 Gy was decreased in a dose-dependent manner (Fig 3), and this phenomenon was similar to that of cellular degranulation in radiation-exposed mast cells (Fig 2).

**Fig 4:**
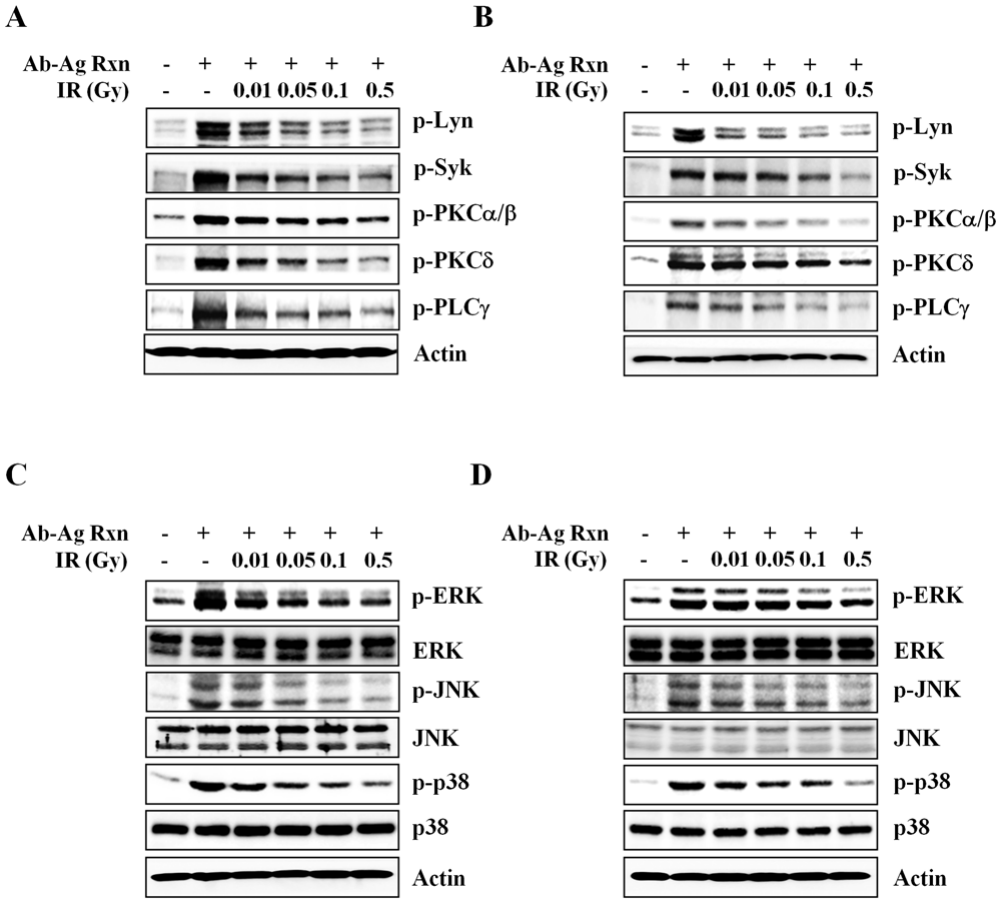
Effects of ionizing radiation on IgE-mediated signal transduction in human mast cells. HMC-1(5C6) (A and C) and LAD2 (B and D) were incubated for 30 min in medium containing human IgE and then stimulated with anti-IgE. IgE-sensitized HMC-1(5C6) and LAD2 cells were exposed to 0–0.5 Gy ionizing radiation and stimulated with anti-IgE. Phosphorylation of signaling molecules was measured with western blotting 5 min (A and B) or 10 min (C and D) after the addition of anti-IgE. Actin was used as an internal standard. Ab-Ag Rxn: antigen-antibody reaction, IR: irradiation.

Cross-linking of FcεRI receptors by the antigen-antibody reaction induces phosphorylation within approximately 1 sec on tyrosine residues of several proteins and results in mast cell degranulation. Therefore, we examined FcεRI-mediated signaling molecules. Notably, within 30 sec to approximately 1 min of antigen stimulation, phosphorylation of several upstream and downstream signaling molecules (Lyn, Syk, protein kinase C [PKC], and PLC-γ) was increased and maintained for 30 min (data not shown). Concomitant with decrease in the level of [Ca^2+^]_i_ induced by ionizing radiation, inhibition of phosphorylation of Lyn, Syk, PKC, and PLC-γ in irradiated human mast cells (HMC-1(5C6) and LAD2 cells) was seen upon stimulation with IgE and antigen in a dose-dependent manner (Fig 4A and 4B).

MAP kinase subfamilies (ERK, P38, JNK) were also activated by IgE-mediated activation of mast cells, resulting in cytokine production. Therefore, we examined the levels of phosphorylated ERK, p38, and JNK. Phosphorylation of all MAP kinase subfamilies was induced within approximately 1 min following mast cell activation and was inhibited by low-dose ionizing radiation (Fig 4C and 4D).

**Fig 5:**
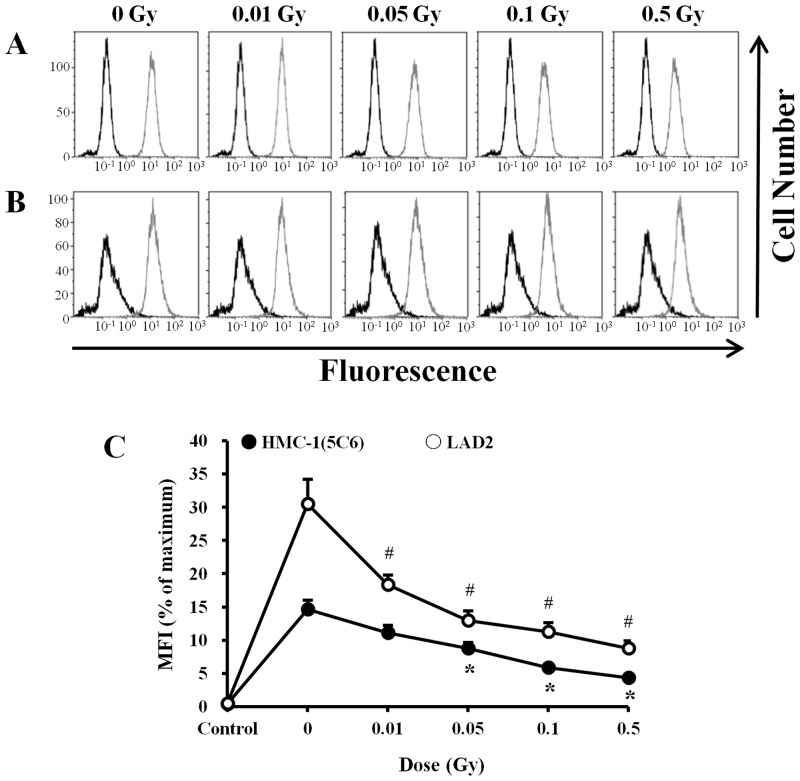
Effects of ionizing radiation on FcεRI expression in human mast cells. HMC-1(5C6) (A) and LAD2 (B) cells were incubated for 30 min in medium containing human IgE and exposed to 0–0.5 Gy ionizing radiation. Thirty minutes after irradiation, cells were incubated with FITC-conjugated human anti-IgE for 30 min. FcεRI expression was evaluated with flow cytometry. (C) The mean fluorescence intensity (MFI) is expressed as the mean ± SEM relative to the mean maximal MFI (100%). Data are representative of three experiments. *p<0.05, #p < 0.05 versus the corresponding control group of HMC-1(5C6) and LAD2 cells, respectively.

In a previous study [20], we confirmed that low-dose ionizing radiation reduces the expression of FcεRI receptors and decreases the release of mediators and cytokines by reducing FcεRI receptor internalization in RBL-2H3 mast cells. Therefore, we examined whether ionizing radiation altered FcεRI expression in human mast cells. As seen in our previous study, ionizing radiation also decreased the expression of FcεRI receptors in human mast cells (HMC-1(5C6) and LAD2 cells) (Fig 5).

### Inhibitory effect of ionizing radiation on IgE-mediated passive cutaneous anaphylaxis and the late-phase cutaneous reaction

**Fig 6:**
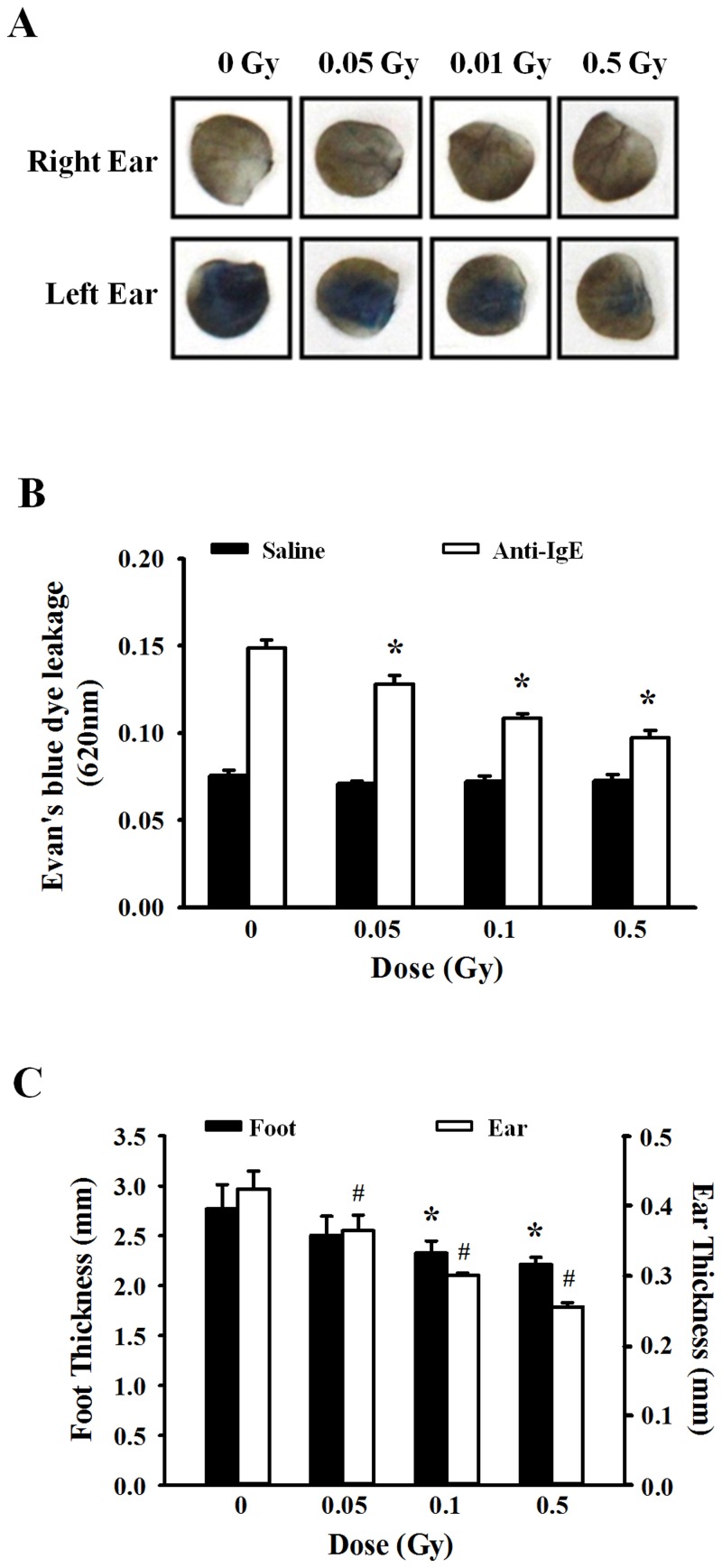
Effects of ionizing radiation in IgE-mediated passive cutaneous anaphylaxis and the late-phase cutaneous model. Anti-DNP (20 ng) was injected intradermally in the left ear, whereas the right ear was injected with saline as a control. After 24 h, mice were irradiated with 0–0.5 Gy before injection of 100 μg DNP-HAS containing Evan’s blue dye via the tail vein. (A) After 30 min, ear punches (8 mm) from both ears were collected and used for extraction of the Evan’s blue dye (B). The optical density was measured at 620 nm. (C) Mice were passively sensitized by i.v. injection of 2 μg anti-DNP IgE. After 24 h, mice were irradiated with 0–0.5 Gy before 20 μl of 0.3% DNFB in acetone/olive oil (4:1) was applied epicutaneously to both sides of the left hind paw and left ear. Acetone/olive oil (20 μl) was applied to the right ear and right hind paw as a control. After 24 h, the thicknesses of the ear and hind paw were measured using a digital microcaliper. Data represent the mean ± SEM from five independent experiments. *p<0.05, #p < 0.05 versus the corresponding control group of foot and ear, respectively.

To evaluate whether ionizing radiation had a similar effect *in vivo*, IgE-mediated passive cutaneous anaphylaxis and the late-phase cutaneous reaction were examined in mouse models. First, to examine the effect of ionizing radiation on IgE-mediated passive cutaneous anaphylaxis in an immediate-type allergic model, the left ears of mice were passively sensitized with 20 ng anti-DNP IgE, and the right ears were injected with saline as a control. Twenty-four hours later, non-irradiated or irradiated mice were injected with 100 μg DNP-HSA containing Evans blue dye via the tail vein. Thirty minutes later, tissues from both ears were collected for extraction of Evans blue dye to determine vascular permeability. Increased vascular permeability was observed in non-irradiated mice. On the contrary, vascular permeability was decreased in irradiated mice (Fig 6A). Fig 6B shows a histogram of the absorbance data after extracting the Evan’s blue dye. These results were similar to those observed in the mediator release experimental data (Fig 2). Finally, as seen Fig 6A and 6B, significant inhibition of the anaphylactic response was noted in ionizing radiation-treated mice (p < 0.05), demonstrating the significant functional impact of low-dose ionizing radiation on antigen-induced anaphylaxis *in vivo*.

We next examined the development of FcεRI-dependent late-phase cutaneous reactions in IgE-sensitized mice and radiation-treated mice. Mice were sensitized with anti-DNP IgE 24 h before a solution of 0.3% DNFB was applied epicutaneously to the ear skin or hind paw. Fig 6C showed that swelling responses occurred only in the IgE-injected left ears and hind paws of mice. No significant increase in ear or hind paw thickness was apparent in the mice treated with vehicle (acetone/olive oil, 4:1) during the experiment. However, low-dose ionizing radiation significantly inhibited the increased in IgE/antigen-specific edema as measured by tissue thickness.

**Fig 7:**
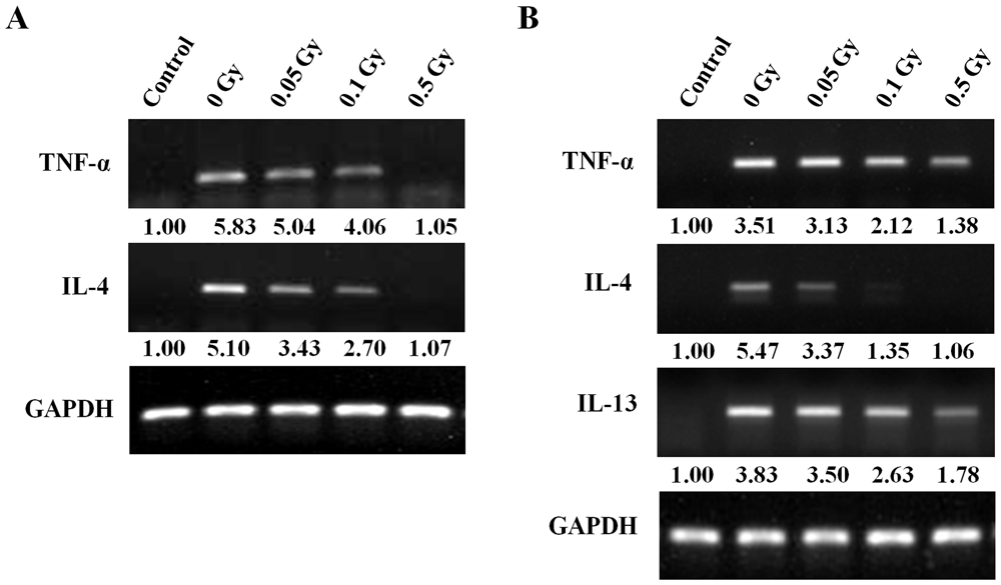
The changes in cytokine mRNA in the ear tissues. TNF-α, IL-4, IL-13 mRNA was determined by RT-PCR in ear tissue of passive cutaneous anaphylaxis (A) and late-phase cutaneous mouse model (B). Semi-quantitative data was indicated as the fold. Each number represents the mean of three experiments.

The production of mediator and cytokines by mast cells may be important in the pathogenesis and inflammatory disease. Therefore, we examined the mRNA level of a various inflammatory cytokines (TNF-α, IL-4, IL-13) in the ear tissue of passive cutaneous anaphylaxis and late-phase cutaneous mouse model (Fig 7). Strong expression of inflammatory cytokines mRNA was found in ear tissue of cutaneous mouse model, inhibited by irradiation.

### Effect of ionizing radiation on histological changes in ear tissue

**Fig 8:**
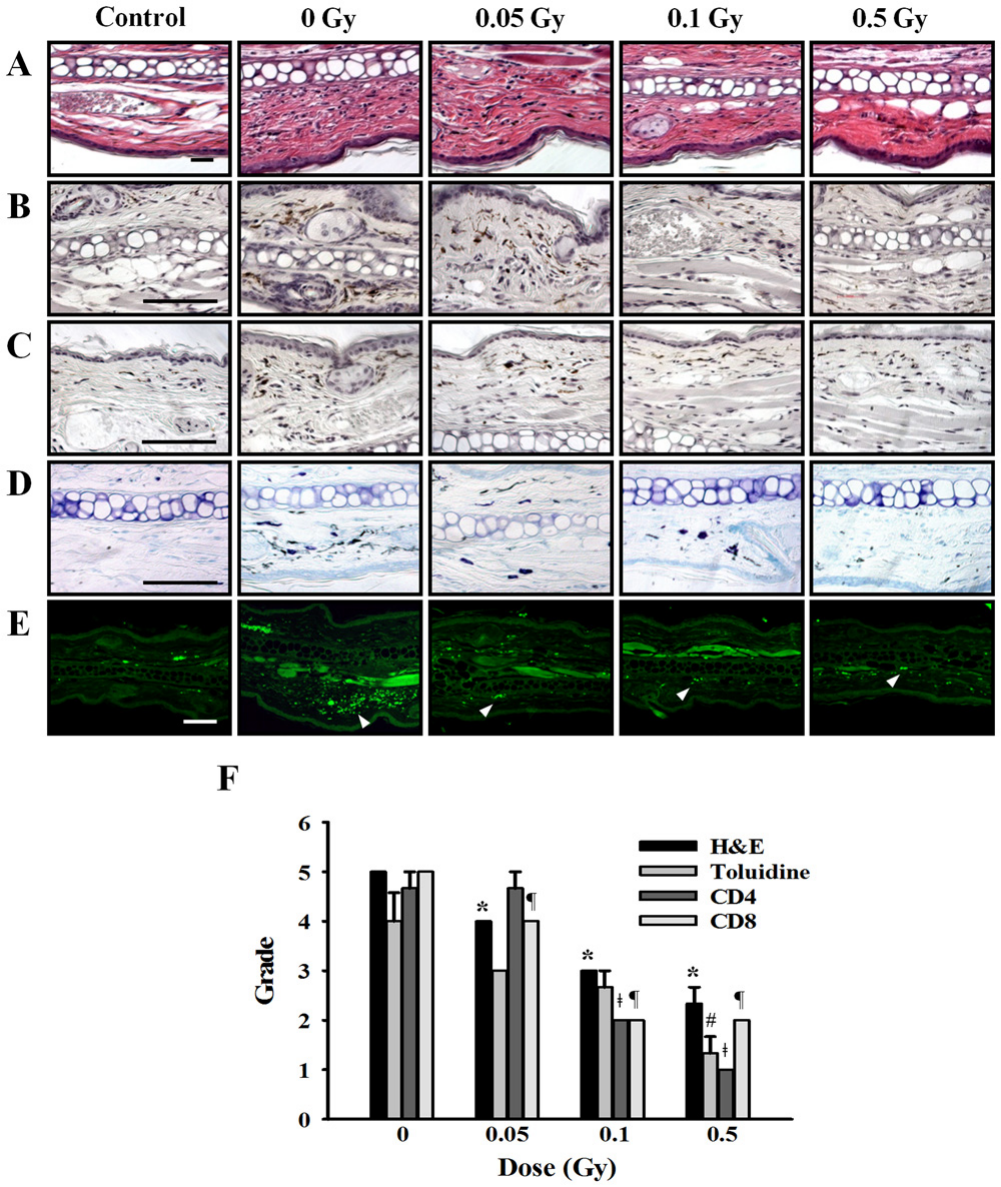
Effects of ionizing radiation on histological changes in ear tissue of passive cutaneous anaphylaxis model. Anti-DNP (20 ng) was injected intradermally in the left ear, whereas the right ear was injected with saline as a control. After 24 h, mice were irradiated with 0–0.5 Gy before injection of 100 μg DNP-HAS via the tail vein. After 5 hr, sections of ear tissue were stained with H&E (A), immunohistochemistry for CD4 (B) and CD8 (C), toluidine blue (D), and FcεRI expression (E) observed at 400× magnification. Histological appearance was score for the presence of infiltrating cells (F). Bars = 100 μm. * p < 0.05, # p < 0.05, ǂ p < 0.05, ¶ p < 0.05 versus the corresponding 0 Gy of H&E, Toluidine, CD4, and CD8, respectively.

**Fig 9:**
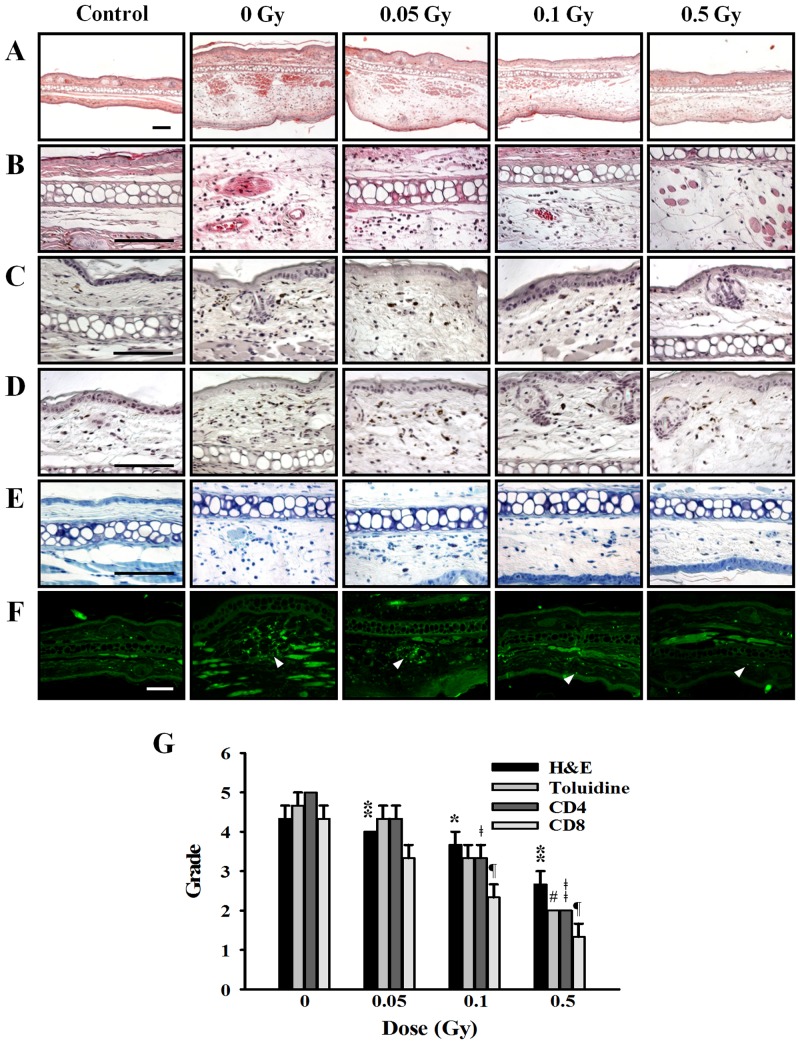
Effects of ionizing radiation on histological changes in ear tissues of late-phase cutaneous reaction model. (A) Mice were passively sensitized by i.v. injection of 2 μg anti-DNP IgE. After 24 h, mice were irradiated with 0–0.5 Gy before 2 μl of 0.3% DNFB in acetone/olive oil (4:1) was applied epicutaneously to the sides of the left ear. Acetone/olive oil (20 μl) was applied to the right ear as a control. After 24 h, sections of ear tissue were stained with H&E (A and B), immunohistochemistry for CD4 (C) and CD8 (D), toluidine blue (E), and FcεRI expression (F) observed at 100× or 400× magnification. Histological appearance was score for the presence of infiltrating cells (G). Bars = 100 μm. * p < 0.05, # p < 0.05, ǂ p < 0.05, ¶ p < 0.05 versus the corresponding 0 Gy of H&E, Toluidine, CD4, and CD8, respectively.

Figs 8 and 9 showed the histological changes in the ear skin lesions of passive cutaneous anaphylaxis and late-phase cutaneous reaction mice. Marked infiltration of inflammatory cells (Figs 8A and 9A and 9B; H&E staining), eosinophils and neutrophils, CD4 cells (Figs 8B and 9C), CD8 cells (Figs 8C and 9D) as well as mast cells (Figs 8D and 9E; Toluidine staining) were evident in the passive cutaneous anaphylaxis and late-phase cutaneous reaction mice ear tissue. However, in the irradiated group, infiltration of inflammatory cells, CD4 cells, CD8 cells, and mast cell was reduced in a dose-dependent manner. There is no histological change in control group mice. In the late-phase cutaneous mice model, we also observed the hypertrophy of epidermis in positive group it was reduced by irradiation (Fig 9A). Histological appearances were scored for the presence of inflammation cells (Figs 8F and 9G).

In the mast cell system, we showed that ionizing radiation inhibit the expression of FcεRI receptor. Therefore, we examined the FcεRI expression in the mouse ear tissue of passive cutaneous anaphylaxis and late-phase cutaneous reaction model. Generally, FcεRI receptor expression was increased in passive cutaneous anaphylaxis and late-phase cutaneous reaction model and reduced by irradiation (Figs 8E and 9F).

## Discussion

Previous studies, we showed that low-dose ionizing radiation inhibits mast cell activation in RBL-2H3 mast cells [20]. However, to predict radiation health effects in humans, we should understand how cellular responses occurring in a multicellular organism are integrated to produce a systemic response. Therefore, in this study, we examined the effects of low-dose ionizing radiation in human mast cell lines (HMC-1(5C6) and LAD2 cells) and cutaneous mouse models because the goal of this study was to analyze the system as a whole, rather than individual parts.

Mast cells play an important role in allergic reactions, but studying human mast cells has been limited by the absence of suitable long-term cultures. Recently, HMC-1(5C6) and LAD2 cells were established and extensively used as surrogates for non-transformed primary human mast cells [21, 22, 25]. Therefore, we used HMC-1(5C6) and LAD2 cells as human mast cell lines.

We first examined the cell viability affected by ionizing radiation in HMC-1(5C6) and LAD2 cells because cell viability is commonly used a criterion to determine cytotoxicity. Cell viability was reduced by 1 Gy irradiation in both human mast cell lines. However, LAD2 cells were more resistant than HMC-1(5C6) cells to ionizing radiation-induced cell cytotoxicity. This may be because LAD2 cells are a slowing-growing (the number of cells doubles in less than 10 to 14 days) and highly differentiated human mast cell line.

Mast cells may be activated by a number of stimuli that are both FcεRI dependent and FcεRI independent. Activation through various receptors leads to distinct signaling pathways [8]. After IgE-mediated activation, mast cells may immediately secrete granule-associated mediators and generate lipid-derived substances that induce immediate allergic inflammation and anaphylactic reactions *in vivo*. Cytokine production is not merely an acute response to IgE-mediated activation but persists for many hours after mast cell activation and may contribute to chronic inflammation and the development of late-phase allergic reactions [26, 27]. Therefore, we examined mediator release and cytokine production induced by ionizing radiation with IgE-mediated human mast cell activation. Low-dose ionizing radiation suppressed production of the granule-associated mediators, histamine and β-hexosaminidase and the inflammatory cytokine TNF-α. Although we also measured production of interleukin-4 and interleukin-8, which are major inflammatory cytokines, because mast cells produce a wide variety of cytokines after IgE-mediated activation, we could not detect these cytokines. On the contrary cell system, we can detect allergy associated cytokines such as TNF-α, IL-4, IL-13 by RT-PCR *in vivo* system (Fig 7). In the both of passive cutaneous anaphylaxis and late-phase cutaneous mouse model, mRNA level of inflammatory cytokines was increased, it was suppressed by irradiation in a dose-dependently manner. These results may explaine that low-dose ionizing radiation suppressed infiltration of inflammatory cells as inhibiting cytokine production in both of mast cell-mediated immediated- and late-phase cutaneous moue model and resulted in anti-allergic effect.

The phosphorylation of FcεRI receptor-dependent tyrosine kinases (Lyn, Syk, PLCγ, PKC) and MAP kinases (ERK, p38, JNK) is involved in mediator release and cytokine production. Therefore, we examined the phosphorylation of signaling molecules following FcεRI-mediated activation and found that phosphorylation of these kinases was inhibited by ionizing radiation in a dose-dependent manner (Fig 4). This phenomenon was caused by decrease in binding affinity between FcεRI receptor and IgE through FcεRI receptor expression reduction. We also examined the FcεRI receptor expression in the ear tissue of mouse model. The infiltration of mast cells was increased in cutaneous model and reduced in the irradiated group. Although it is hard to show the decreased of FcεRI receptor expression in each of the mast cell in vivo, in the progressing study, we got a data that ionizing radiation induced cytoskeletal rearrangement and inhibition of mast cell migration (preparing manuscript) in the mast cell system. Therefore, we thought that ionizing radiation induced decreasing of binding affinity between IgE and FcεRI receptor and inhibition of mast cell migration through cytoskeletal rearrangement.

We also tested our hypothesis using an animal model system because low-dose radiation suppresses allergic reaction via IgE-dependent mast cell activation in various mast cell systems (human mast cells and RBL-2H3 cells). We hypothesized that radiation may distinctly suppress passive cutaneous anaphylaxis due to decreased mediator release and the late-phase cutaneous allergic reaction due to decreased cytokine production because low-dose ionizing radiation of ≤0.5 Gy inhibited mediator release and cytokine production.

An immediate-type allergic reaction was induced a day after i.v. injection of the antigen and Evan’s blue dye. The extravasation of Evan’s blue dye, which is indicative of vascular leakage resulting from mast cell activation and anaphylactic response, was analyzed 30 min after the induction of passive cutaneous anaphylaxis. In patients with allergy, an immediate-phase reaction occurs within 60 min of allergen challenge and is followed by a late-phase reaction, which appears after 3 to 48 h [28]. The late-phase reaction is characterized by infiltration of inflammatory cells and an increase in vasopermeability of various tissues including the skin, lungs, nose, and eyes. The late-phase reaction is of interest because of its similarity to the clinical manifestation of chronic allergic disease [29, 30]. Histologically, the late phase is characterized by edema and mixed cellular infiltration, which is predominantly lymphocytic but also contains eosinophils, neutrophils, and basophils. In the ear skin lesions of the mouse model of passive cutaneous anaphylaxis and late-phase cutaneous model, high numbers of inflammatory cells, CD4 cells, CD8 cells, and mast cells were observed and these patterns were decreased by ionizing radiation (Figs 8 and 9).

To examine the genetic toxicity in the mouse model, we performed a micronucleus test because the cell toxicity response can differ among organisms following ionizing radiation. The micronucleus test is one of the most widely applied short-term tests in genetic toxicology [31]. We found no significant difference (p > 0.05) among the mice regarding the micronucleus frequency in either the irradiated (~0.5 Gy) or the control group. However, the micronucleus frequency was, as expected, significantly higher (p < 0.05) in the 1-Gy irradiated mouse group than the control group (data not shown).

Therefore, in this study, we suggest that low-dose ionizing radiation (≤0.5 Gy) can inhibit mast cell-mediated immediate-type and delayed-type allergic reactions *in vivo* and *in vitro* without inducing cellular or genetic toxicity. Until now, because few researchers have extensively studied the effects of low-dose ionizing radiation on allergic reaction resulting from mast cell activation, these findings through systemic investigation are very meaningful.
